# Growth hormone therapy after hematopoietic cell transplantation in childhood: a nationwide survey and longitudinal cohort study

**DOI:** 10.3389/fendo.2026.1831711

**Published:** 2026-05-20

**Authors:** Maiko Shimomura, Hiroshi Kawaguchi, Eisuke Inoue, Reiko Kagawa, Satoshi Okada, Shohei Yamamoto, Maho Sato, Kana Washio, Miho Ashiarai, Shoji Saito, Akira Hayakawa, Yasuo Ejima, Hiroshi Fuji, Shuichi Ozono, Hirotoshi Sakaguchi, Katsuyoshi Koh, Koji Kato, Katsutsugu Umeda

**Affiliations:** 1 Department of Pediatrics, Hiroshima University Hospital, Hiroshima, Japan; 2 Department of Pediatrics, Hiroshima Red Cross Hospital and Atomic-bomb Survivors Hospital, Hiroshima, Japan; 3 Showa Medical University Research Administration Center, Showa Medical University, Tokyo, Japan; 4 Department of Pediatrics, Tokai University School of Medicine, Isehara, Japan; 5 Department of Hematology/Oncology, Osaka Women’s and Children’s Hospital, Osaka, Japan; 6 Department of Pediatrics, Okayama University Hospital, Okayama, Japan; 7 Department of Pediatrics, St. Luke’s International Hospital, Tokyo, Japan; 8 Department of Pediatrics, Shinshu University School of Medicine, Matsumoto, Japan; 9 Department of Palliative Care Medicine, Yodogawa Christian Hospital, Osaka, Japan; 10 Department of Radiology, Dokkyo Medical University, Tochigi, Japan; 11 Department of Radiation Oncology, Chiba Nishi General Hospital, Chiba, Japan; 12 Department of Pediatrics and Child Health, Kurume University School of Medicine, Kurume, Japan; 13 Children’s Cancer Center, National Center for Child Health and Development, Tokyo, Japan; 14 Department of Hematology/Oncology, Saitama Children’s Medical Center, Saitama, Japan; 15 Central Japan Cord Blood Bank, Seto, Japan; 16 Department of Pediatrics, Faculty of Medical Sciences, University of Fukui, Fukui, Japan

**Keywords:** growth hormone therapy, height outcomes, hematopoietic cell transplantation, late effects, short stature, total body irradiation

## Abstract

**Background:**

Growth impairment is a major late effect in survivors of childhood hematopoietic cell transplantation (HCT); however, the long-term effectiveness and response patterns of growth hormone (GH) therapy after HCT remain unclear. Thus, this study aimed to evaluate the effectiveness of GH therapy and clinical factors associated with GH response in patients who underwent HCT during childhood and were diagnosed with short stature and to further examine long-term response patterns among those receiving GH therapy.

**Methods:**

We conducted a nationwide, multicenter retrospective cohort study of childhood HCT survivors with post-transplant short stature. Height outcomes were evaluated using final adult height standard deviation scores (SDS) and changes in SDS (ΔSDS). Factors associated with height outcomes and GH responsiveness were analyzed using multivariable regression models, and longitudinal growth trajectories after GH initiation were assessed at 1 year, 5 years, and final height.

**Results:**

Among 171 patients with available final height data, 58 received GH therapy. GH-treated patients showed significantly greater improvement in final height SDS and ΔSDS than untreated patients, although growth responses were heterogeneous. Total body irradiation (TBI), HCT before 5 years of age, and chronic graft-versus-host disease were independently associated with poorer height outcomes. Factors associated with GH responsiveness differed by transplant type: absence of TBI and female sex were favorable in allogeneic HCT. Regarding complications, GH therapy was not associated with an increased risk of slipped capital femoral epiphysis, secondary malignancies, or relapse of the primary disease.

**Conclusions:**

GH therapy improves height outcomes after childhood HCT, but responses vary and are strongly influenced by transplant-related toxicity and long-term complications. To improve height outcomes, optimizing GH therapy and developing strategies for patients expected to respond poorly remain important challenges.

## 1 Introduction

Hematopoietic cell transplantation (HCT) is an established curative treatment for children with various malignant and non-malignant disorders, and the number of long-term survivors continues to increase (1, 2). With improving survival rates, endocrine late effects such as impaired linear growth have become significant drivers of long-term morbidity in pediatric HCT survivors (3, 4). Growth hormone (GH) deficiency and impaired height gain are common after HCT, especially among children exposed to total body irradiation (TBI), cranial irradiation, or intensive conditioning regimens (4, 5). More than 30-50% of children receiving TBI-based conditioning develop GH deficiency within several years after HCT, and even patients without overt GH deficiency may experience reduced growth velocity owing to transplant-related growth plate and endocrine disruption (5, 6).

Radiation-related hypothalamic–pituitary injury is a major etiological mechanism of GH deficiency after HCT (5, 7); however, multiple additional transplant-specific factors, such as gonadal dysfunction, chronic graft-versus-host disease (GVHD), inflammation, corticosteroid exposure, and direct damage to the epiphyseal growth plates, collectively contribute to impaired post-transplant growth (3, 5, 7). Consequently, growth impairment after HCT is multifactorial in nature and differs from classical GH deficiency observed in individuals without cancer (3, 7).

GH replacement therapy is recommended for childhood cancer and HCT survivors with biochemically confirmed GH deficiency, and its safety profile has been supported by large international cohorts and registry-based studies (2, 5, 8). Nonetheless, the magnitude and durability of GH responsiveness in HCT survivors highly vary. Although several small-scale studies have reported that GH therapy can improve height standard deviation score (SDS) over 1–2 years (9, 10), long-term catch-up growth to the target height range remains challenging for a considerable proportion of survivors (5, 8, 10).

Additionally, classical predictors of better GH response, such as early initiation of GH therapy and female sex, are derived from non-transplant GH deficiency cohorts, and whether they apply to HCT survivors whose growth plates may have diminished proliferative potential and altered maturation owing to prior oncological therapies and systemic inflammation remains unclear (11–13).

Importantly, most existing evidence has focused either on short-term growth velocity or final adult stature (14, 15). Long-term GH effectiveness and distinct response trajectories, such as sustained versus transient catch-up growth, have not been adequately characterized among pediatric HCT survivors (14, 16). Therefore, we aimed to evaluate the effectiveness of GH therapy and clinical factors associated with GH response in patients who underwent HCT during childhood and were diagnosed with short stature and to further examine long-term response patterns among those receiving GH therapy.

## 2 Materials and methods

### 2.1 Study design and data collection

This retrospective study collected clinical data from medical records of patients with short stature after HCT at 55 institutions participating in the Japan Children’s Cancer Group (JCCG). The list of participating institutions that contributed to the questionnaire but are not included as coauthors is provided in Appendix SA1. This study was conducted jointly by the Transplantation and Cellular Therapy Committee and the Long-term Follow-up Committee of the JCCG. This study was approved by the Ethics Committee of Hiroshima University, which served as the central institutional review board, and the current study was conducted under a centralized ethical review process (approval no. E2024-0176).

Candidate patients were selected according to the following criteria: (1) diagnosis of a hematologic malignancy or solid tumor at a JCCG-participating institution between January 1990 and December 2018; (2) receipt of autologous or allogeneic HCT at ≤15 years; (3) age 15–39 years at the time of study registration; and (4) survival for at least 2 years after HCT with short stature (≤ -2.0 SDS) at that time. Patients with underlying conditions associated with short stature, such as congenital anomalies or chromosomal abnormalities, were excluded.

In total, 268 patients were enrolled in this study. Of these, two patients born small for gestational age and 53 patients who already had short stature prior to HCT were excluded, resulting in a final study population of 213 patients. The effects of GH therapy were evaluated in 171 of these patients for whom final adult height data were available. The incidence of complications, including slipped capital femoral epiphysis, secondary malignancies, and relapse of the primary disease, was compared between the GH-treated (n = 72) and non-GH-treated (n = 141) groups. GH deficiency was diagnosed based on stimulation testing as follows: when two or more GH stimulation tests were performed, GH deficiency was defined as a peak GH level of < 6 ng/mL in each test. In cases with evident intracranial organic lesions (e.g., pituitary or hypothalamic tumors, history of surgery or radiotherapy, or congenital malformations), GH deficiency was defined as a peak GH level of < 6 ng/mL on a single stimulation test, in accordance with criteria commonly used in Japan (17–19). Final height was defined as the height measured after epiphyseal closure, when linear growth had essentially ceased. Insulin-like growth factor-1 (IGF-1) z-scores were calculated based on Japanese reference data (20). Pubertal onset was defined as Tanner stage ≥ 2, based on genital development in boys and breast development in girls. Bone age was assessed using left hand and wrist radiographs according to the Greulich and Pyle method (21), based on information obtained from medical records. Delayed bone age was defined as a difference of > 1 year between bone age and chronological age. Concomitant steroid use was defined as systemic corticosteroid administration (oral or intravenous), including methylprednisolone and prednisolone, at a cumulative prednisone-equivalent dose (22) of ≥ 0.3 mg/kg/day for at least 6 months. Comorbidities were defined as follows: thyroid and gonadal dysfunction were recorded when therapeutic intervention was required; cardiac dysfunction was defined as a left ventricular ejection fraction < 50%; pulmonary dysfunction was defined based on abnormalities detected by pulmonary function testing; and precocious puberty was recorded regardless of treatment status. According to previous reports, myeloablative conditioning was classified as a regimen, including 5 Gy of TBI, total abdominal radiation (TAI), or total lymphoid irradiation (TLI) as a single fraction, TBI/TAI/TLI of ≥ 8 Gy in fractionated doses, or oral or intravenous busulfan at > 8 mg/kg. Other conditioning regimens were classified as reduced-intensity conditioning (23, 24).

### 2.2 Statistical analysis

Continuous variables are summarized as medians with interquartile ranges (IQRs), and categorical variables as numbers and percentages. Height outcomes were evaluated using height SDSs. Growth response was defined as the difference between height SDS at the diagnosis of short stature and final adult height SDS (ΔSDS). The two independent groups were compared using the Mann–Whitney U test because ΔSDS was not normally distributed. Categorical variables were compared using Fisher’s exact test. Univariate analyses were initially performed to identify factors associated with ΔSDS. For height outcome analyses, variables with p < 0.1 and clinically established determinants of post-transplant growth were included in multivariate models (25–28). In contrast, analyses of GH treatment response were limited to variables with p < 0.1 because of the smaller sample size. These variables included GH therapy, TBI, history of brain tumor, steroid exposure, age at transplantation, sex, craniospinal irradiation (CSI), thyroid dysfunction, gonadal dysfunction, and chronic GVHD. Prespecified subgroup analyses were performed in patients who received GH therapy, stratified according to transplant type (allogeneic vs. autologous). In GH-treated patients, additional multivariate linear regression analyses were performed to evaluate the effects of age at GH initiation (≥ 10 vs. < 10 years), sex, and TBI. An interaction term between age at GH initiation and sex was included to assess potential effect modification. The association between the interval from transplantation to initiation of GH therapy and ΔSDS was evaluated using univariable linear regression. Pubertal status at GH initiation (Tanner stage ≥ 2 vs < 2) was analyzed using the Mann–Whitney U test in sex- and transplant-specific subgroups. Longitudinal changes in ΔSDS after initiation of GH therapy (1 year, 5 years, and final height) were analyzed using the Friedman test, followed by *post hoc* Wilcoxon signed-rank tests. The 5-year time point was omitted for patients with a follow-up duration of < 5 years. All statistical analyses were performed using EZR (version 1.61, Saitama Medical Center, Jichi Medical University, Saitama, Japan) (29), a graphical user interface for R. Spaghetti plots depicting individual longitudinal ΔSDS trajectories were generated using Python (version 3.10.12). All hypothesis tests were two-sided, and a p-value < 0.05 was considered statistically significant.

## 3 Results

### 3.1 Patient characteristics

In total, 171 patients with available final height data were included in the analysis (**Table 1**). Among them, 58 patients received GH therapy and 113 did not. The median age at study registration was 23 years, and the ages at diagnosis and at HCT were comparable between the GH and non-GH groups. Sex distribution and the number of HCTs were comparable between the two groups. The GH group included a higher proportion of brain tumor survivors and autologous transplant recipients than the non-GH group. Exposure to TBI was less frequent, whereas CSI was more common in the GH group than in the non-GH group. Data on pubertal status and bone age were not available for all patients; therefore, analyses of these variables were based on available data and cautiously interpreted. Thyroid dysfunction was more prevalent among patients receiving GH therapy than among those not receiving GH therapy, whereas other comorbidities, pubertal status, and delayed bone age were comparable between groups. Of the 58 GH-treated patients with available final height data, GH deficiency was confirmed in 40 patients, including severe and non-severe cases (**Supplementary Table 1**). GH therapy was initiated at a median age of 11.4 years, approximately 6 years after transplantation. The median duration of GH therapy was 5.3 (IQR, 3.0–7.2) years. The median durations were 5.9 (IQR, 3.15–8.10) years in allogeneic HCT recipients and 4.9 (IQR, 2.83–6.63) years in autologous HCT recipients (p = 0.253).

**Table 1:** Characteristics of the 171 patients included in the study.

Variable	Factors	Overall	(n=171)	GH	(n=58)	non-GH	(n=113)	*p* value
Age at study registration (years)	Median (IQR)	23	(19.5-26.4)	23	(18.2-26.4)	23	(19.9-26.4)	0.823
Age at diagnosis (years)	Median (IQR)	4.1	(1.9-6.8)	2.9	(1.35-6.4)	4.2	(2.7-7.9)	0.206
Age at HCT (years)	Median (IQR)	4.7	(2.7-7.7)	4.2	(2.3-6.9)	4.8	(2.8-8.8)	0.291
Follow-up duration (years)	Median (IQR)	18.2	(14.4-22.2)	18.0	(14.9-21.6)	18.3	(13.8-22.3)	0.944
Sex, n (%)	Male	89	(52.0)	27	(46.6)	62	(54.9)	0.334
	Female	82	(48.0)	31	(53.4)	51	(45.1)	
Diagnosis, n (%)	Hematologic malignancies	116	(67.8)	30	(51.7)	86	(76.1)	< 0.01
	Solid tumors (excluding brain tumors)	38	(22.2)	15	(25.9)	23	(20.4)	
	Brain tumor	17	(9.9)	13	(22.4)	4	(3.5)	
Type of transplant, n (%)	Allogeneic	116	(67.8)	31	(53.4)	85	(75.2)	< 0.01
	Autologous	55	(32.2)	27	(46.6)	28	(24.8)	
Number of HCT, n (%)	1	124	(72.5)	41	(70.7)	83	(73.5)	0.902
	2	41	(24.0)	15	(25.9)	26	(23.0)	
	3	6	(3.5)	2	(3.4)	4	(3.5)	
	4	0	(0.0)	0	(0.0)	0	(0.0)	
RT	TBI (> 8 Gy)	101	(59.1)	28	(48.3)	73	(64.6)	0.049
	CSI	14	(8.1)	12	(20.7)	2	(0.9)	< 0.01
	CRT (without spinal irradiation)	7	(4.1)	3	(5.2)	4	(3.5)	0.69
	Other craniospinal irradiation	9	(5.3)	6	(10.3)	3	(2.7)	0.063
	No	40	(23.3)	9	(15.5)	31	(27.4)	0.08
Conditioning chemotherapy	BU > 8mg/kg	29	(16.9)	9	(15.5)	20	(17.6)	0.831
	MEC	10	(5.8)	4	(6.9)	6	(7.1)	0.717
	TEPA	17	(9.9)	11	(18.9)	6	(5.3)	0.004
	Reduced-intensity conditioning	8	(4.7)	3	(5.2)	5	(4.4)	0.659
	Other (including TBI-based MAC)	107	(62.6)	31	(53.4)	76	(67.2)	0.07
Steroids	Yes	32	(18.7)	10	(17.2)	22	(19.5)	0.935
	No	135	(78.9)	47	(81.0)	88	(77.9)	
	Unknown	4	(3.4)	1	(1.7)	3	(2.7)	
Puberty*								
Tanner stage ≧ 2	Yes No Unknown	39 66 66	(22.8) (38.6) (38.6)	12 31 15	(20.7) (53.4) (25.9)	27 35 51	(23.9) (31.0) (45.1)	0.15
Delayed bone age*								
> 1 years behind	Yes No Unknown	37 26 108	(21.6) (15.2) (63.2)	20 10 28	(34.4) (17.2) (48.2)	17 16 80	(15.0) (14.2) (70.8)	0.306
Comorbidities*	Thyroid dysfunction	53	(31.0)	30	(51.7)	23	(20.3)	< 0.01
	Cardiac dysfunction	9	(5.3)	5	(8.6)	4	(3.5)	0.169
	Gonadal dysfunction	109	(63.7)	40	(69.0)	69	(61.1)	0.401
	Pulmonary dysfunction	35	(20.5)	12	(20.7)	23	(20.4)	1
	Precocious puberty	10	(5.8)	6	(10.3)	4	(3.5)	0.09

*Variables were assessed at the time of short stature diagnosis or at initiation of GH therapy. Data on the timing of GH initiation are provided in **Supplementary Table 1**.

Percentages are calculated using the total number of patients in each group as the denominator. Missing data are indicated where applicable.

GH, Growth hormone; IQR, Interquartile range; HCT, Hematopoietic cell transplantation; RT, Radiation therapy; TBI, Total body irradiation; CSI, Craniospinal irradiation; CRT, Cranial radiotherapy; BU, Busulfan; MEC, Melphalan/Etoposide/Carboplatin; TEPA, Thiotepa; MAC, myeloablative conditioning.

### 3.2 Factors associated with final height in patients with short stature after HCT

**Figure 1:**
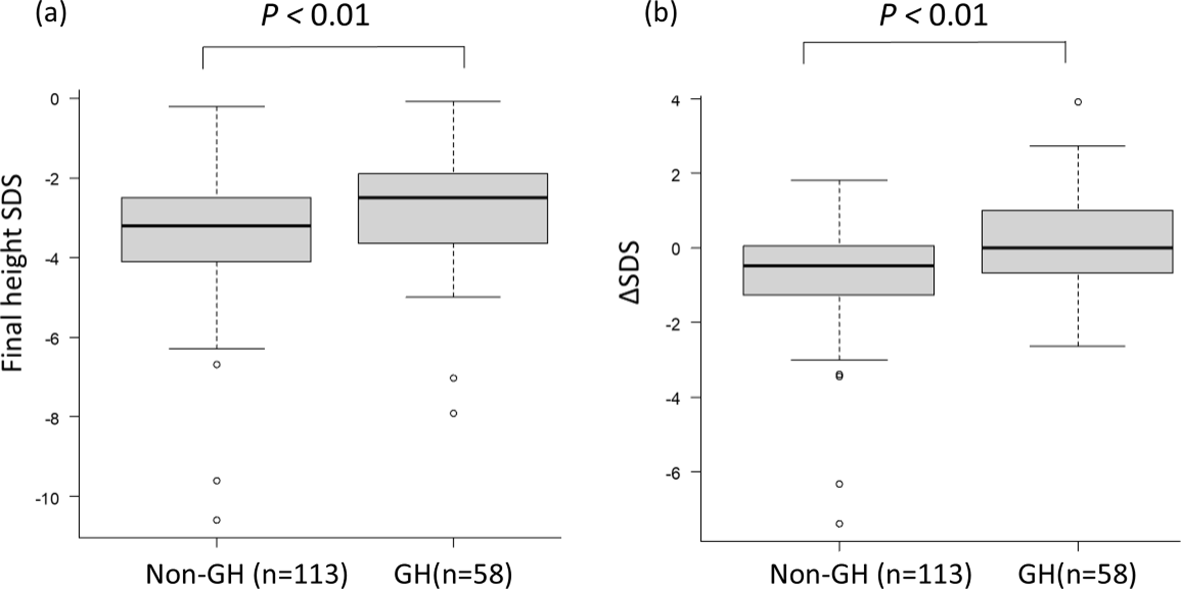
Comparison of final height SDS and ΔSDS from the time of short stature diagnosis to final height between patients with and without GH therapy. **(A)** Final height SDS and **(B)** ΔSDS from short stature diagnosis to final height were compared between patients with and without GH therapy using the Mann–Whitney U test.

Final height SDS and the difference between final height SDS and height SDS at the time of short stature diagnosis (ΔSDS) were compared between patients with and without GH therapy. GH therapy was associated with a significantly greater improvement in both outcomes (p < 0.01) (**Figure 1**). To identify factors influencing post-transplant short stature, univariate and multivariable analyses were performed including GH therapy and other clinically relevant variables (**Supplementary Table 2**; **Table 2**). Consequently, GH therapy and older age at HCT (≥ 5 years) were associated with an increase in final height SDS, whereas TBI-containing regimens and chronic GVHD were associated with a decrease in final height SDS.

**Table 2:** Multivariable linear regression analysis of factors associated with final height SDS.

Variable	Adjusted mean ΔSDS (no)	Adjusted mean ΔSDS (yes)	Difference (yes–no)	95% CI (lower–upper)	*p* value
GH therapy (yes vs no)	−1.23	−0.29	0.94	0.49 to 1.38	< 0.001
TBI (yes vs no)	−0.38	−1.14	−0.76	−1.21 to −0.31	0.001
Age at HCT (≥ 5 vs < 5 years)	−1.13	−0.39	0.75	0.34 to 1.15	< 0.001
Chronic GVHD (yes vs no)	−0.46	−1.06	−0.60	−1.15 to −0.06	0.031
CSI (yes vs no)	−0.68	−0.84	−0.16	−1.38 to 1.05	0.79
Steroid use (yes vs no)	−0.51	−1.01	−0.50	−1.09 to 0.09	0.098
Thyroid dysfunction (yes vs no)	−0.67	−0.86	−0.19	−0.65 to 0.27	0.41
Gonadal dysfunction (yes vs no)	−0.62	−0.90	−0.28	−0.71 to 0.14	0.19
Sex (Male vs Female)	−0.62	−0.90	−0.28	−0.69 to 0.13	0.17
Brain tumor (yes vs no)	−0.51	−1.01	−0.49	−1.66 to 0.67	0.41

SDS, Standard deviation score; GH, Growth hormone; TBI, Total body irradiation; HCT, Hematopoietic cell transplantation; GVHD, Graft-versus-host disease; CSI, Craniospinal irradiation.

### 3.3 Factors associated with the response to growth hormone therapy after HCT

Among the 58 patients who received GH therapy and had available final height data, analyses were performed to identify factors associated with ΔSDS and height outcomes. In the overall multivariable analysis, absence of TBI, initiation of GH therapy at age ≥ 10 years, and female sex were identified as favorable factors (**Table 3**).

**Table 3:** Multivariable linear regression analysis of factors associated with the response to GH therapy.

Cohort	Variable	Adjusted mean ΔSDS (no)	Adjusted mean ΔSDS (yes)	Difference (yes–no)	95% CI (lower–upper)	*p* value
All	TBI (yes vs no)	0.525	–0.793	–1.32	–1.92 to –0.74	< 0.001
	Age at GH initiation ≥ 10 years	–0.566	0.297	0.863	0.29 to 1.54	0.005
	Sex (male vs female)	0.225	–0.493	–0.72	–1.37 to –0.19	0.011
	Gonadal dysfunction	–0.307	0.039	0.346	–0.31 to 0.99	0.293
Allogenic HCT	TBI (yes vs no)	1.466	–0.525	–1.99	–3.41 to –0.57	0.008
	Sex (male vs female)	0.937	0.004	–0.93	–1.76 to –0.10	0.029
	Conditioning (MAC vs RIC)	0.435	0.506	0.07	–1.56 to1.71	0.929
Autologous HCT	TBI (yes vs no)	1.208	0.356	−0.85	−1.92 to 0.21	0.11
	Steroid (yes vs no)	−0.212	1.776	1.99	−0.37 to 4.35	0.095
	Age at GH initiation ≥ 10 years	0.244	1.320	1.08	0.16 to 1.99	0.024

GH, Growth hormone; SDS, Standard deviation score; TBI, Total bodey irradiation; HCT, Hematopoietic cell transplantation.

In the allogeneic HCT cohort, absence of TBI and female sex were independently associated with better height outcomes, whereas in the autologous HCT cohort, initiation of GH therapy at ≥ 10 years was a favorable factor in the multivariable analysis. Results of the univariable analyses are presented in **Supplementary Table 3**. Age at HCT was positively correlated with age at initiation of GH therapy (Spearman’s rank correlation coefficient ρ = 0.444, p = 0.0231). Patients who initiated GH therapy at ≥ 10 years tended to have a higher prevalence of severe GH deficiency compared with those who initiated therapy at age < 10 years. However, this difference did not reach statistical significance (45.8% vs 25.0%, p = 0.3).

### 3.4 Longitudinal patterns of height response to GH therapy

**Figure 2:**
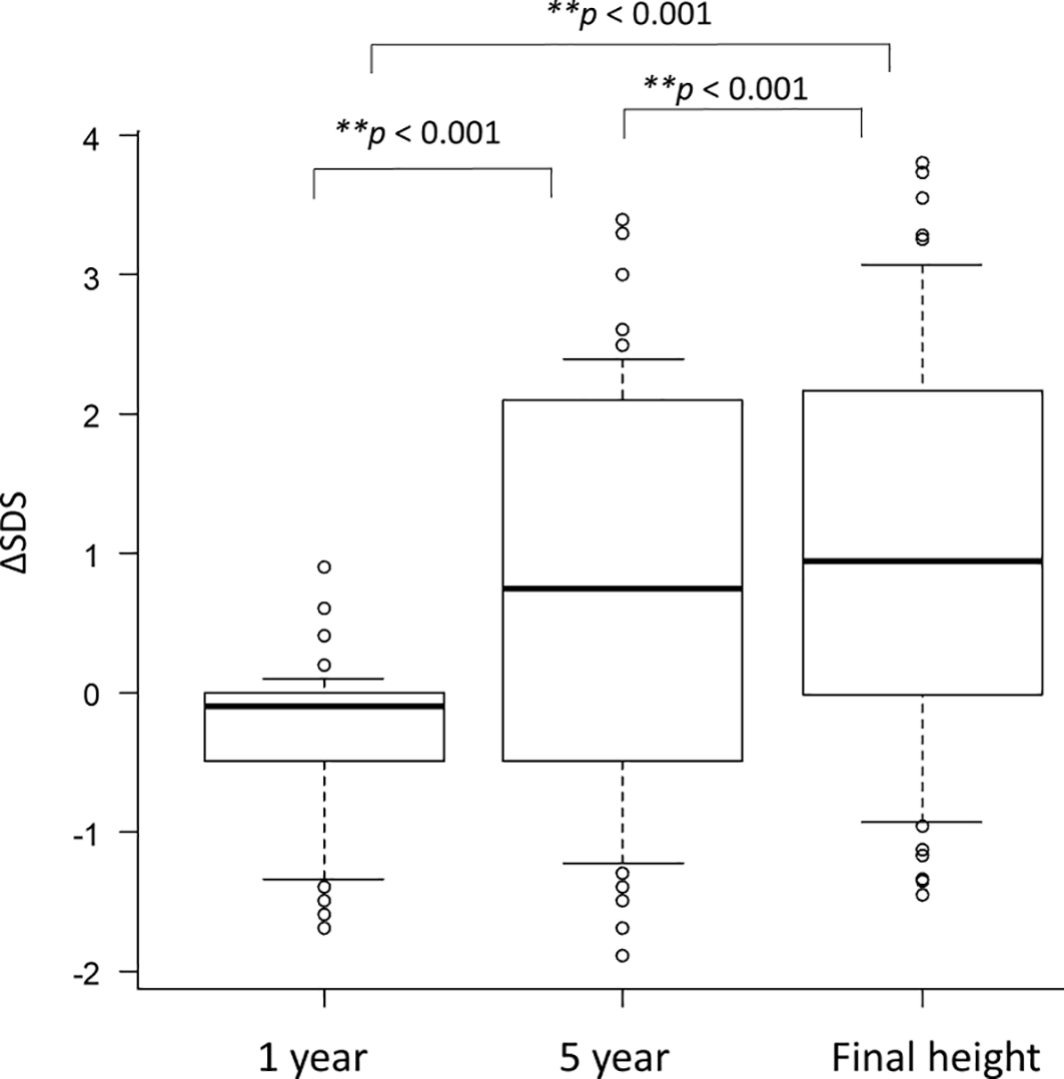
Longitudinal changes in ΔSDS among GH-treated patients. ΔSDS in GH-treated patients was compared by years after initiation of GH therapy. Overall differences across the three time points—1 year after initiation of GH therapy (1), 5 years after initiation of GH therapy (2), and at final height assessment (3)—were assessed using the Friedman test (χ²(2) = 31.85, *p* < 0.001). Pairwise comparisons between time points 1 and 2, 2 and 3, and 3 and 1 were performed using the Wilcoxon signed-rank test. **p < 0.001.

**Figure 3:**
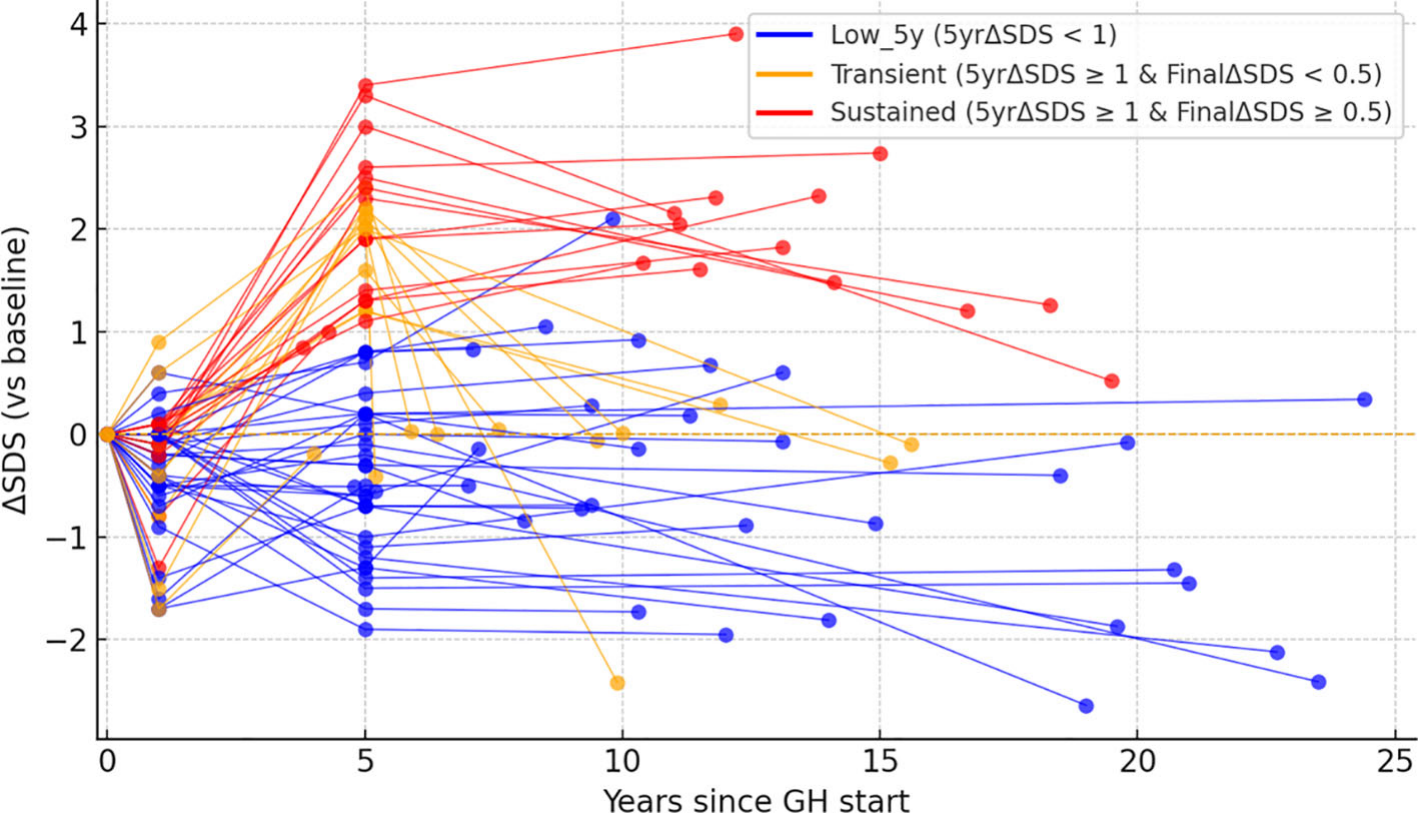
Longitudinal trajectories of GH-treated patients. GH-treated patients were evaluated in 57 cases after excluding one case with unknown observation period. Cases with a 5-year ΔSDS < 1 are shown in blue (n = 31); those with a 5-year ΔSDS ≥ 1 but a final height ΔSDS < 0.5 are shown in orange (n = 11), and cases with a 5-year ΔSDS ≥ 1 and a final height ΔSDS ≥ 0.5 are shown as red lines (n = 15).

ΔSDS was longitudinally compared in patients who received GH therapy. Changes in ΔSDS at 1 year after GH initiation, at 5 years, and at the time of final height assessment demonstrated a pattern distinct from that reported in the general population, with greater improvement observed at 5 years than in the first year and further improvement at final height, as shown in **Figure 2** (Friedman test, χ² (2) = 31.85, p < 0.001). Furthermore, individual longitudinal changes in ΔSDS are shown in **Figure 3** to complement the comparison of median ΔSDS at each time point. The patients were classified into three groups according to their growth response: Low_5y (5-year ΔSDS < 1, n = 31), Transient (5-year ΔSDS ≥ 1 and final ΔSDS < 0.5, n = 11), and Sustained (5-year ΔSDS ≥ 1 and final ΔSDS ≥ 0.5, n = 15). Each line represents an individual patient. Among the 57 evaluable patients, factors associated with achieving a ΔSDS ≥ 1 at 5 years after GH initiation were examined. For analyses of longitudinal GH treatment response, we examined clinically relevant and those variables identified in the univariable analyses of height outcomes (**Supplementary Table 2**), including TBI, history of brain tumor, steroid exposure, age at HCT and at GH initiation, sex, CSI, thyroid dysfunction, gonadal dysfunction, and chronic GVHD. Absence of TBI was significantly associated with a favorable 5-year height response (p < 0.01), whereas initiation of GH therapy at age ≥ 10 years showed a borderline association (p = 0.05). Sex was not associated with achieving ΔSDS ≥ 1 at 5 years (**Supplementary Table 4****).** Among the 26 patients who achieved a ΔSDS ≥ 1 at 5 years, factors associated with sustained height gain to final height were further evaluated. Female sex and initiation of GH therapy at ≥10 years tended to be more frequent among patients with sustained improvement (final ΔSDS ≥ 0.5), although these differences did not reach statistical significance (**Supplementary Table 5****).** Overall, even among patients with a favorable early response at 5 years, long-term trajectories were heterogeneous, highlighting distinct patterns of sustained, transient, and poor growth responses following GH therapy.

### 3.5 Complications associated with GH therapy

We compared complication rates between patients with and without GH therapy in a cohort of 213 patients, including individuals for whom final height data were unavailable (**Supplementary Tables 6**, **7**). These analyses were performed using univariable comparisons. The median follow-up duration for this cohort was 16.7 years (IQR, 11.3–20.9). TBI exposure was significantly associated with secondary malignancies (p < 0.01). Importantly, GH therapy was not associated with an increased risk of slipped capital femoral epiphysis, secondary malignancies, or relapse of the primary disease However, these findings should be interpreted with caution and considered exploratory given the limited number of events.

## 4 Discussion

In this study, we evaluated the effectiveness and long-term response patterns of GH therapy in survivors who underwent HCT during childhood and subsequently developed short stature. Using final adult height data, we demonstrated that GH therapy was associated with improved height outcomes after HCT. Importantly, longitudinal analyses revealed heterogeneous growth trajectories, including sustained, transient, and poor responses, highlighting the complexity of post-transplant growth impairment and its management.

Consistent with previous studies, GH therapy was associated with significantly greater improvement in final height SDS and ΔSDS compared with patients who did not receive GH therapy (10, 11). Earlier reports have primarily focused on short-term growth velocity or height gain within the first 1–2 years after GH initiation and often demonstrated modest but variable benefits. Our findings extend this evidence by showing that GH therapy was associated with measurable improvement even at the level of final adult height in our cohort (30–32). Nevertheless, the magnitude of growth recovery remained limited in many patients, supporting the concept that post-HCT growth impairment is multifactorial. Transplant-related factors, such as radiation exposure, chronic inflammation, endocrine dysfunction, and skeletal toxicity likely constrain the biological potential for catch-up growth, even in the presence of GH replacement (33). TBI emerged as one of the strongest negative predictors of height outcomes and GH responsiveness. This observation is consistent with previous reports, showing that intensive conditioning with TBI is associated with long-term growth deceleration and impaired final height in children undergoing HCT (30, 31). As indicated in the multivariate analysis (**Table 3**), the absence of TBI was significantly associated with greater ΔSDS at final height even among GH-treated patients, suggesting that GH therapy cannot fully overcome radiation-induced growth limitations. Chronic GVHD was also independently associated with poorer height outcomes. Persistent inflammation, prolonged corticosteroid exposure, impaired nutrition, and metabolic complications related to chronic GVHD are associated with adverse growth and cardiometabolic profiles in childhood HCT survivors (12, 33). Taken together, these findings underscore that preservation of growth potential after HCT depends not only on endocrine replacement therapy but also on minimizing transplant-related toxicity and late effects (31–33).

Importantly, factors associated with GH responsiveness differed between allogeneic and autologous HCT recipients. Absence of TBI and female sex were independently associated with better growth response in the allogeneic HCT cohort, whereas initiation of GH therapy at age ≥ 10 years was the primary favorable factor in the autologous HCT cohort. Moreover, notably, the proportion of patients exposed to TBI differed between the allogeneic and autologous HCT cohorts (**Supplementary Table 3**), reflecting differences in underlying diseases and conditioning strategies. These differences likely reflect distinct pathophysiological backgrounds between transplant types. After allogeneic HCT, growth impairment is influenced not only by pre-transplant therapies but also by post-transplant immune-mediated factors, including chronic GVHD, delayed immune reconstitution, and long-term steroid exposure (25, 31). Such ongoing insults may attenuate the impact of age-related factors and limit sustained growth recovery.

In contrast, autologous HCT recipients are generally less affected by immune-mediated complications, and their growth outcomes may be more directly determined by pre-transplant treatment intensity and residual skeletal growth potential, as suggested in previous long-term series of pediatric HCT survivors (25, 31). The finding that GH initiation at age ≥ 10 years was associated with a more favorable growth response, particularly in the autologous HCT cohort, appears counterintuitive, as earlier initiation of GH therapy is typically associated with better outcomes in both non-transplant populations with GH deficiency and post-HCT cohorts (10, 11, 14, 34). Although not statistically significant, a higher proportion of patients with severe GH deficiency was observed among those who initiated GH therapy at age ≥ 10 years, which may partly explain the trend toward a more favorable GH response in this group (35, 36). Several considerations are important when interpreting this result. First, this analysis inherently included only patients who survived after transplantation and subsequently received GH therapy, introducing survivor bias. Second, GH therapy was not randomly assigned but initiated according to clinical judgment, which may have differed across institutions, raising the possibility of indication bias. Moreover, given the limited number of cases and the wide variability in ΔSDS, the adverse impact of younger age at HCT may have been underestimated (1, 8, 24, 29–32). In contrast, our findings indicate that, in contrast to previous reports, clinically meaningful growth benefits can still be expected even when GH therapy is initiated at an older age, depending on the individual patient’s residual growth potential and the duration of continued treatment. Female sex was a favorable factor for GH response in the overall and allogeneic HCT cohorts and tended to be associated with sustained height gain to final height. This finding is consistent with observations in non-transplant GH deficiency cohorts, in which females often demonstrate greater GH sensitivity and more durable growth responses than males (14, 32, 34, 37). Potential mechanisms include differences in sex steroid interactions with the GH–IGF-1 axis, pubertal growth patterns, and epiphyseal maturation (14, 32, 34, 37). However, given the limited sample, these findings should be cautiously interpreted.

The longitudinal evaluation of individual growth trajectories following GH therapy is a major strength of this study. Unlike prior studies focusing on short-term outcomes or final height alone, we demonstrated that growth responses in HCT survivors are heterogeneous over time. Some patients achieved sustained improvement through final height, whereas others showed transient catch-up growth or minimal response. Notably, ΔSDS continued to improve beyond the first year of GH therapy, with greater gains observed at 5 years and at final height. This delayed pattern contrasts with that reported in the general GH-deficient population and may reflect gradual recovery of growth plate function or progressive mitigation of transplant-related systemic insults (14, 15, 31–33). Clinically, these findings suggest that early assessment of GH efficacy may underestimate long-term benefit and that prolonged follow-up is essential when evaluating treatment response in this population.

GH therapy was not associated with an increased risk of slipped capital femoral epiphysis, secondary malignancies, or relapse of the primary disease. Previous large cohort studies evaluating the long-term safety of GH therapy have also reported generally reassuring results, although some concerns regarding long-term mortality have been raised in specific populations (38). In contrast, TBI exposure was significantly associated with secondary malignancies, consistent with the results of previous studies (39–41). Although the observational design and limited number of events and observational period preclude definitive conclusions regarding long-term safety, our findings support the overall safety of GH therapy in carefully selected childhood HCT survivors. This study has several limitations. Its retrospective design introduces potential residual confounding. GH initiation criteria, dosing, and duration were not standardized across institutions, reflecting real-world practice but adding heterogeneity. In addition, some patients received GH therapy without confirmed GH deficiency. Although these cases were few, no apparent differences were observed in final height or Δ height SDS. However, this finding should be interpreted with caution. Only a small number of patients transitioned from daily to once-weekly GH therapy or to adult GH therapy, and these cases were excluded due to insufficient data, likely reflecting the relatively short follow-up period and the retrospective design. As discussed above, selection bias is unavoidable, as only patients referred for GH evaluation and treatment were included. Additionally, information on parental height and genetic growth potential was unavailable.

In conclusion, GH therapy is associated with improved height outcomes in childhood HCT survivors with short stature; however, responses are heterogeneous and strongly influenced by transplant-related factors. Determinants of GH responsiveness appear to differ between allogeneic and autologous HCT, suggesting distinct underlying mechanisms of growth impairment. These findings underscore the need for individualized, long-term management strategies and careful counseling when considering GH therapy in this complex population.

## Data Availability

The raw data supporting the conclusions of this article will be made available by the authors, without undue reservation.
