# Supplementary material for: Growth hormone therapy after hematopoietic cell transplantation in childhood: a nationwide survey and longitudinal cohort study
Source: Front Endocrinol (Lausanne). 2026 May 20;17:1831711. doi: 10.3389/fendo.2026.1831711 (PMC13229625; doi:10.3389/fendo.2026.1831711)
Supplement: Supplementary file 1 [file DataSheet1.docx]

| **Variables** | **n** | **Median** | **(IQR)** |
| --- | --- | --- | --- |
| Age at start of GH therapy (years) | 58 | 11.4 | (9.2 to 13.2) |
| Interval from HCT to GH initiation (years) | 58 | 5.8 | (4.3 to 8.47) |
| Duration of GH therapy (years) | 58 | 5.3 | (3.0 to 7.2) |
| Baseline IGF-1 (ng/mL) | 49/58 | 135 | (92 to 194) |
| Baseline IGF-1 (z-score) | 49/58 | -1.8 | (-2.7 to -0.8) |
| Height SDS at initiation | 58 | -3 | (-3.6 to -2.62) |
| Final height (cm) |  |  |  |
| All | 58 | 147.6 | (143.1 to 154.1) |
| Male | 27 | 151.8 | (146.2 to 155.9) |
| Female | 31 | 143.1 | (138.7 to 146.9) |
| Final height SDS | 58 | -2.5 | (-3.6 to -1.9) |
| Final height ΔSDS | 58 | 0.01 | (-0.55 to 1.0) |
| **Variables** | **n** | **%** |  |
| Tanner stage ≥2 at GH initiation in male | 7/27 | (25.9) |  |
| Tanner stage ≥2 at GH initiation in female | 5/31 | (16.1) |  |
| Patients who underwent GH stimulation testing | 54/58 | (93.1) |  |
| GH deficiency confirmed | 40/54 | (74.1) |  |
| GH deficiency not confirmed | 7/54 | (12.9) |  |
| Unknown | 7/54 | (12.9) |  |
| Severe GHD (Peak GH < 3 ng/mL) | 25/40 | (62.5) |  |
| Non-severe GHD (Peak GH 3–10 ng/mL) | 15/40 | (37.5) |  |
| Switching from daily GH to once-weekly GH therapy | 0/58 | (0.0) |  |
| Concomitant steroid use | 11/57 | (19.3) |  |
| Transition to adult GHD | 2/58 | (3.4) |  |

**Supplementary TABLE 1. Characteristics of the 58 GH-treated patients with available final height data**

GH; Growth hormone, IQR; Interquartile range, HCT; Hematopoietic stem cell transplantation, IGF-1; Insulin-like growth factor 1, SDS; Standard deviation score, GHD; Growth hormone deficiency

**Supplementary TABLE 2. Univariable analysis of factors associated with final height SDS**

| **Factors** | **Variables** | **n** | **median (IQR)** | ***p* value*** |
| --- | --- | --- | --- | --- |
| GH therapy | Yes | 58 | 0.005 (–0.65 to 0.98) | <0.01 |
|  | No | 113 | –0.49 (–1.28 to 0.05) |  |
| Sex | Male | 89 | –0.43 (–1.23 to 0.13) | 0.117 |
|  | Female | 82 | –0.13 (–0.95 to 0.49) |  |
| Age at HCT | ≥5 year | 80 | –0.13 (–0.86 to 0.41) | 0.152 |
|  | <5 year | 91 | –0.45 (–1.42 to 0.20) |  |
| Category | Hematologic malignancies | 116 | –0.43 (–1.23 to 0.20) | 0.146 |
|  | Solid tumors | 55 | –0.07 (–0.76 to 0.35) |  |
| Puberty** | Yes | 39 | –0.41 (–0.86 to 0.07) | 0.631 |
|  | No | 66 | –0.01 (–1.56 to 0.60) |  |
| Delayed bone age** | Yes | 37 | –0.07 (–1.45 to 0.83) | 0.15 |
|  | No | 26 | –0.70 (–1.10 to –0.20) |  |
| Thyroid dysfunction | Yes | 53 | –0.40(–1.63 to 0.20) | 0.403 |
|  | No | 118 | –0.26 (–0.95 to 0.32) |  |
| Cardiac dysfunction | Yes | 9 | –0.40 (–0.89 to 0.35) | 0.989 |
|  | No | 162 | –0.27 (–1.19 to 0.25) |  |
| Gonadal dysfunction | Yes | 109 | –0.40 (–1.32 to 0.38) | 0.663 |
|  | No | 62 | –0.24 (–0.84 to 0.09) |  |
| Pulmonary dysfunction | Yes | 35 | –0.56 (–1.93 to 0.19) | 0.147 |
|  | No | 136 | –0.20 (–0.96 to 0.30) |  |
| Precocious puberty | Yes | 10 | –0.20 (–1.59 to 0.23) | 0.818 |
|  | No | 161 | –0.28 (–1.16 to 0.28) |  |
| Brain tumor | Yes | 17 | 0.03 (–0.54 to 0.67) | 0.05 |
|  | No | 154 | –0.40 (–1.23 to 0.20) |  |
| TBI | Yes | 101 | –0.68 (–1.53 to 0.01) | <0.01 |
|  | No | 70 | 0.00 (–0.54 to 0.70) |  |
| BU | Yes | 29 | –0.56 (–1.24 to 0.92) | 0.959 |
|  | No | 142 | –0.24 (–0.95 to 0.20) |  |
| CSI | Yes | 14 | 0.005 (–0.13 to 0.58) | 0.046 |
|  | No | 157 | –0.27 (–1.17 to 0.28) |  |
| CRT | Yes | 7 | –0.40 (–1.19 to 0.42) | 0.873 |
|  | No | 164 | –0.265 (–1.17 to 0.28) |  |
| Other spinal irradiation | Yes | 9 | –0.51 (–1.70 to 1.48) | 0.795 |
|  | No | 162 | –0.27 (–1.15 to 0.27) |  |
| Transplant type | Allogeneic | 116 | –0.42 (–1.25 to 0.22) | 0.104 |
|  | Autologus | 55 | –0.06 (–0.76 to 0.34) |  |
| Chronic GVHD | Yes | 45 | –0.91 (–1.87 to –0.25) | <0.01 |
|  | No | 126 | –0.06 (–0.78 to 0.40) |  |
| Steroids** | Yes | 33 | –0.91 (–1.75 to –1.32) | <0.01 |
|  | No | 134 | –0.100 (–0.88 to 0.34) |  |

*Mann–Whitney U test

**For variables with missing data (pubertal status, bone age and steroids), univariable analyses were performed using available cases only.

SDS; Standard deviation score, IQR; Interquartile range, GH; Growth hormone, IQR; Interquartile range, TBI; Total body irradiation, BU; Busulfan, CSI; Craniospinal irradiation, CRT; Cranial radiotherapy, GVHD; Graft-versus-host disease

**Supplementary TABLE 3. Univariable analyses of factors associated with the response to GH therapy**

|  | | **All** | | | **Allogenic HCT** | | | **Autologous HCT** | | |  |
| --- | --- | --- | --- | --- | --- | --- | --- | --- | --- | --- | --- |
| **Variables** | **Category** | **n** | **median (range)** | ***p* value** | **n** | **median (range)** | ***p* value** | **n** | **median (range)** | ***p* value** | |
| Sex | Male | 27 | –0.19 (–0.88 to 0.235) | 0.01 | 15 | –0.50 (–1.84 to 0.02) | 0.01 | 12 | –0.03 (–0.72 to 0.32) | 0.3 | |
|  | Female | 31 | 0.52 (–0.21 to 1.64) |  | 16 | 0.59 (–0.13 to 1.21) |  | 15 | 0.52 (–0.32 to 1.71) |  | |
| Age at HCT | ≥5 years | 25 | 0.34 (–0.19 to 0.84) | 0.23 | 16 | 0.095 (–0.24 to 0.94) | 0.318 | 9 | 0.52(–0.06 to 0.67) | 0.322 | |
|  | <5 years | 33 | –0.10 (–1.32 to 1.48) |  | 15 | –0.40 (–1.80 to 1.36) |  | 18 | –0.035 (–0.75 to 1.18) |  | |
| Age at start of GH therapy | ≥10 years | 39 | 0.18(–0.41 to 1.23) | 0.036 | 23 | 0.01(–0.455 to 1.10) | 0.386 | 16 | 0.43(–0.08 to 1.66) | 0.026 | |
|  | <10 years | 19 | –0.28(–1.38 to 0.28) |  | 8 | –0.415(–1.10 to 0.47) |  | 11 | –0.10 (–1.38 to 0.17) |  | |
| Category | HM | 30 | –0.07 (–0.53 to 1.03) | 0.988 | 28 | –0.035 (–0.59 to 1.08) | 0.385 | 2 | –0.08 (–0.09 to –0.07) | 0.627 | |
|  | Solid tumors | 28 | 0.04 (–0.72 to 0.71) |  | 3 | –0.41(–1.18 to –0.03) |  | 25 | 0.05 (–0.69 to 0.84) |  | |
| Puberty | Yes | 12 | –0.06(–0.40 to 0.55) | 0.685 | 8 | –0.16 (–0.40 to 0.05) | 0.392 | 4 | 1.61(0.36 to 2.17) | 0.416 | |
|  | No | 31 | 0.29 (–0.53 to 1.16) |  | 15 | 0.92 (–1.21to 1.46) |  | 16 | 0.29 (–0.14 to 0.67) |  | |
| Delayed bone age | Yes | 20 | –0.08 (–1.54 to 1.21) | 0.779 | 13 | 0.01 (–1.81 to 1.20) | 0.916 | 7 | –0.89 (–1.38 to 1.04) | 0.5 | |
|  | No | 10 | –0.17 (–0.41 to 0.02) |  | 8 | –0.295(–0.43 to –0.04) |  | 2 | –0.005 (–0.03 to 0.02) |  | |
| Steroid* | Yes | 11 | –0.19(–0.61 to 0.62) | 0.686 | 10 | –0.30 (–0.67 to 0.1) | 0.416 | 1 | 2.74(2.74 to 2.74) | 0.076 | |
|  | No | 46 | 0.03 (–0.655 to 0.98) |  | 21 | 0.28 (–0.55 to 1.2) |  | 25 | 0.00 (–0.69 to 0.67) |  | |
| Brain tumor | Yes | 13 | 0.03 (–0.14 to 0.67) | 0.621 | 0 | – | N.A | 13 | 0.03 (–0.14 to 0.67) | 0.65 | |
|  | No | 45 | –0.06 (–0.72 to 1.05) |  | 31 | –0.11 (–0.67 to 1.04) |  | 14 | –0.01 (–0.795 to 1.19) |  | |
| TBI** | Yes | 28 | –0.40 (–1.52 to 0.29) | <0.01 | 22 | –0.34 (–1.51 to 0.26) | <0.01 | 6 | –1.10 (–1.41 to 0.032) | 0.085 | |
|  | No | 30 | 0.56 (–0.09 to 1.78) |  | 9 | 1.67 (1.05 to 2.1) |  | 21 | 0.05 (–0.10 to 0.84) |  | |
| BU** | Yes | 9 | 0.92 (–0.69 to 1.67) | 0.356 | 6 | 1.06 (–0.42 to 1.55) | 0.448 | 3 | 0.05 (–0.32 to 1.18) | 0.689 | |
|  | No | 49 | -0.06 (–0.55 to 0.83) |  | 25 | –0.14 (–0.55 to 0.83) |  | 24 | 0.015 (–0.59 to 0.71) |  | |
| CSI | Yes | 12 | 0.005 (–0.11 to 0.61) | 0.645 | 1 | 0.01 (0.01-0.01) | 0.968 | 11 | 0.00 (–0.12 to 0.63) | 0.753 | |
|  | No | 46 | –0.015 (–0.83 to 1.16) |  | 30 | –0.11 (–0.67 to 1.03) |  | 16 | 0.04 (–0.12 to 0.63) |  | |
| CRT | Yes | 3 | –0.08 (–0.24 to 0.42) | 1 | 3 | –0.08 (–0.24 to 0.42) | 0.925 |  | – |  | |
|  | No | 55 | 0.01 (–0.71 to 1.02) |  | 28 | –0.07 (–0.75 to 1.09) |  |  | – |  | |
| Other spinal irradiation | Yes | 6 | 0.765 (–0.37 to 1.98) | 0.451 | 0 | – | N.A | 6 | 0.765 (–0.37 to 1.98) | 0.476 | |
|  | No | 52 | ‐0.03 (–0.69 to 0.86) |  | 31 | –0.11 (–0.67 to 1.04) |  | 21 | 0.00 (–0.69 to 0.60) |  | |
| Type of HCT | Allogeneic | 31 | –0.08 (–0.63 to 1.02) | 0.726 |  | – |  |  | – |  | |
|  | Autologous | 27 | 0.03 (–0.60 to 0.75) |  |  | – |  |  | – |  | |
| Conditioning | MAC |  | – |  | 25 | –0.14 (–0.87 to 0.83) | 0.046 |  | – |  | |
|  | RIC |  | – |  | 6 | 1.55 (0.12 to 2.55) |  |  | – |  | |
| MAC | TBI |  | – |  | 19 | –0.28 (–1.22 to 0.23) | <0.01 |  | – |  | |
|  | BU |  | – |  | 3 | 1.67 (1.43 to 1.89) |  |  | – |  | |
| Number of HCT | 1 | 28 | –0.06 (–0.50 to 1.05) | 0.484 | 21 | –0.14 (–0.14 to 1.05) | 0.983 | 7 | 0.00 (–1.40 to 0.17) | 0.263 | |
|  | ≥2 | 30 | 0.05 (–0.87 to 0.83) |  | 10 | 0.23 (–0.79 to 0.89) |  | 20 | 0.185 (–0.23 to 1.00) |  | |
| Chronic GVHD | Yes | 12 | –0.165 (–0.75 to 0.21) | 0.318 | 12 | –0.165 (–0.76 to 0.21) | 0.484 | 0 | – | N.A | |
|  | No | 46 | 0.04 (–0.54 to 1.03) |  | 19 | 0.35 (–0.48 to 1.12) |  | 27 | 0.03 (–0.6 to 0.755) |  | |
| Thyroid dysfunction | Yes | 30 | –0.025 (–0.41 to 0.79) | 0.957 | 13 | –0.08 (–0.41 to 0.28) | 0.489 | 17 | 0.05 (–0.14 to 1.48) | 0.386 | |
|  | No | 28 | 0.015 (–0.75 to 1.09) |  | 18 | 0.365 (–0.68 to 1.25) |  | 10 | 0.015 (–0.75 to 0.32) |  | |
| Gonadal dysfunction | Yes | 40 | 0.315 (–0.55 to 1.27) | 0.09 | 21 | 0.280 (–0.40 to1.20) | 0.124 | 19 | 0.34 (–0.60 to 1.16) | 0.449 | |
|  | No | 18 | –0.19 (–0.76 to 0.045) |  | 10 | –0.455 (–1.43 to 0.07) |  | 8 | –0.03 (–0.28 to 0.035) |  | |

* Information was missing for one patient.

**TBI/BU exposure includes reduced-intensity conditioning doses.

GH; Growth hormone, HCT; Hematopoietic cell transplantation, TBI; Total body irradiation, BU; Busulfan, CSI; Craniospinal irradiation CRT; Cranial radiotherapy, MAC; Myeloablative conditioning, RIC; Reduced-intensity conditioning, GVHD; Graft-versus-host disease, N.A; Not Applicable

**Supplementary TABLE 4. Univariable analysis of factors associated with achieving ΔSDS ≥1 at 5 years after GH initiation**

|  |  | **ΔSDS≧1** | |  | | **ΔSDS<1** | |  | ***p* value** |
| --- | --- | --- | --- | --- | --- | --- | --- | --- | --- |
|  |  | **n** | **%** | | | **n** | **%** | |  |
| Sex | Male | 12 | 46.2 | | 15 | | 48.4 | | 1 |
|  | Female | 14 | 53.8 | | 16 | | 51.6 | |  |
| Age at HCT | ≥5 year | 11 | 42.3 | | 13 | | 41.9 | | 1 |
|  | <5 year | 15 | 57.7 | | 18 | | 58.1 | |  |
| Age at start of GH therapy | ≥10 years | 21 | 80.7 | | 17 | | 54.8 | | 0.05 |
|  | <10 years | 5 | 19.2 | | 14 | | 45.2 | |  |
| Brain tumor | Yes | 8 | 30.8 | | 5 | | 16.1 | | 0.22 |
|  | No | 18 | 69.2 | | 26 | | 83.9 | |  |
| TBI | Yes | 7 | 26.9 | | 20 | | 64.5 | | <0.01 |
|  | No | 19 | 73.1 | | 11 | | 35.5 | |  |
| CSI | Yes | 7 | 26.9 | | 5 | | 16.1 | | 0.349 |
|  | No | 19 | 73.1 | | 26 | | 83.9 | |  |
| Thyroid dysfunction | Yes | 13 | 50 | | 16 | | 51.6 | | 1 |
|  | No | 13 | 50 | | 15 | | 48.4 | |  |
| Gonadal dysfunction | Yes | 19 | 73.1 | | 20 | | 64.5 | | 0.574 |
|  | No | 7 | 26.9 | | 11 | | 35.5 | |  |
| Chronic GVHD | Yes | 5 | 19.2 | | 7 | | 22.6 | | 1 |
|  | No | 21 | 80.8 | | 24 | | 77.4 | |  |
| Steroids* | Yes | 5 | 19.2 | | 6 | | 20 | | 1 |
|  | No | 21 | 80.8 | | 24 | | 80 | |  |

*Uunivariable analyses were performed using available cases only.

SDS; Standard deviation score, GH; Growth hormone, HCT; Hematopoietic Cell Transplantation, TBI; Total body irradiation, CSI; Craniospinal irradiation, GVHD; Graft-versus-host disease

**Supplementary TABLE 5. Univariable analysis of factors associated with final height among patients who achieved ΔSDS ≥1 at 5 years after GH initiation**

|  |  | **ΔSDS≧0.5** | |  | | **ΔSDS<0.5** | |  | ***p* value** |
| --- | --- | --- | --- | --- | --- | --- | --- | --- | --- |
|  |  | **n** | **%** | | | **n** | **%** | |  |
| Sex | Male | 7 | 63.6 | | 5 | | 33.3 | | 0.233 |
|  | Female | 4 | 36.3 | | 10 | | 66.7 | |  |
| Age at HCT | ≥5 year | 4 | 36.4 | | 7 | | 46.7 | | 0.701 |
|  | <5 year | 7 | 63.6 | | 8 | | 53.3 | |  |
| Age at start of GH therapy | ≥10 years | 9 | 81.8 | | 8 | | 53.3 | | 0.128 |
|  | <10 years | 2 | 18.2 | | 7 | | 46.7 | |  |
| Brain tumor | Yes | 4 | 36.3 | | 4 | | 26.7 | | 0.683 |
|  | No | 7 | 63.6 | | 11 | | 73.3 | |  |
| TBI | Yes | 4 | 36.3 | | 3 | | 20 | | 0.407 |
|  | No | 7 | 63.6 | | 12 | | 80 | |  |
| CSI | Yes | 3 | 27.3 | | 4 | | 26.7 | | 1 |
|  | No | 8 | 72.7 | | 11 | | 73.3 | |  |
| Thyroid dysfunction | Yes | 6 | 54.5 | | 7 | | 46.7 | | 1 |
|  | No | 5 | 45.5 | | 8 | | 53.3 | |  |
| Gonadal dysfunction | Yes | 10 | 90.9 | | 9 | | 60 | | 0.178 |
|  | No | 1 | 9.1 | | 6 | | 40 | |  |
| Chronic GVHD | Yes | 2 | 18.2 | | 3 | | 20 | | 1 |
|  | No | 9 | 81.8 | | 12 | | 80 | |  |
| Steroids | Yes | 2 | 18.2 | | 3 | | 20 | | 1 |
|  | No | 9 | 81.8 | | 12 | | 80 | |  |

SDS; Standard deviation score, GH; Growth hormone, HCT; Hematopoietic Cell Transplantation, TBI; Total body irradiation, CSI; Craniospinal irradiation, GVHD; Graft-versus-host disease

**Supplementary TABLE 6. Characteristics of the 213 patients included in the study**

| **Variable** | **Factors** | **Overall** | **(n=213)** | **GH** | **(n=72)** | **non-GH** | **(n=141)** | ***p* value** |
| --- | --- | --- | --- | --- | --- | --- | --- | --- |
| **Age at study registration (years)** | Median (IQR) | 21.7 | (16.8-26) | 21.8 | (16.8-24.9) | 21.7 | (16.8-26.1) | 0.945 |
| **Age at diagnosis (years)** | Median (IQR) | 3.8 | (1.8-6.5) | 2.9 | (1.25-6.4) | 4 | (1.9-7.0) | 0.095 |
| **Age at HCT (years)** | Median (IQR) | 4.8 | (2.3-7.5) | 3.9 | (2.0-13.3) | 4.8 | (2.7-7.2) | 0.273 |
| **Follow-up duration (years)** | Median (IQR) | 16.7 | (11.3-20.9) | 16.2 | (13.1-20.8) | 16.8 | (10.8-21.0) | 0.586 |
| **Sex, n (%)** | Male | 112 | (52.6) | 36 | (50.0) | 76 | (53.9) | 0.664 |
|  | Female | 101 | (47.4) | 36 | (50.0) | 65 | (46.1) |  |
| **Diagnosis, n (%)** | Hematologic malignancies | 144 | (67.6) | 38 | (52.8) | 106 | (75.2) | <0.01 |
|  | Solid tumors (excluding brain tumors) | 45 | (21.1) | 18 | (25.0) | 27 | (19.1) |  |
|  | Brain tumor | 24 | (11.3) | 16 | (22.2) | 8 | (5.7) | <0.01 |
| **Type of transplant, n (%)** | Allogeneic | 144 | (67.6) | 39 | (54.2) | 105 | (74.5) | <0.01 |
|  | Autologous | 69 | (32.4) | 33 | (45.8) | 36 | (25.5) |  |
| **Number of HCT, n (%)** | 1 | 150 | (70.4) | 50 | (69.4) | 100 | (70.9) | 0.88 |
|  | 2 | 54 | (25.4) | 20 | (27.8) | 34 | (24.1) |  |
|  | 3 | 7 | (3.3) | 2 | (2.8) | 5 | (3.5) |  |
|  | 4 | 2 | (0.9) | 0 | (0.0) | 2 | (1.4) |  |
| **RT** | TBI (>8Gy) | 115 | (53.9) | 32 | (44.4) | 83 | (58.9) | 0.058 |
|  | CSI | 20 | (9.3) | 14 | (19.4) | 6 | (4.2) | <0.01 |
|  | CRT (without spinal irradiation) | 9 | (4.2) | 3 | (4.2) | 6 | (4.3) | 1 |
|  | Other craniospinal irradiation | 13 | (6.1) | 8 | (11.1) | 5 | (3.5) | 0.037 |
|  | No | 56 | (26.5) | 15 | (20.9) | 41 | (29.1) | 0.67 |
| **Conditioning chemotherapy** | BU > 8mg/kg | 44 | (20.7) | 14 | (19.4) | 30 | (21.3) | 0.859 |
|  | MEC | 13 | (6.1) | 5 | (6.9) | 8 | (7.1) | 0.717 |
|  | TEPA | 17 | (7.9) | 11 | (15.3) | 6 | (4.3) | 0.008 |
|  | Reduced-intensity conditioning | 14 | (6.5) | 4 | (5.6) | 10 | (7.1) | 0.78 |
|  | Other (including TBI-based MAC) | 125 | (58.6) | 38 | (52.8) | 87 | (61.7) | 0.24 |
| **Steroids** | Yes | 39 | (18.3) | 13 | (18.1) | 26 | (18.4) | 1 |
|  | No | 170 | (79.8) | 58 | (80.6) | 112 | (79.4) |  |
|  | Unknown | 4 | (1.8) | 1 | (1.4) | 3 | (2.1) |  |
| **Puberty*** |  |  |  |  |  |  |  |  |
| Tanner stage ≧ 2 | Yes  No  Unknown | 47  90  76 | (22.1)  (42.2)  (35.7) | 14  42  16 | (19.4)  (58.3)  (22.2) | 33  48  60 | (23.4)  (34.0)  (42.6) | 0.11 |
| **Delayed bone age*** |  |  |  |  |  |  |  |  |
| >1 years behind | Yes  No  Unknown | 43  41  129 | (20.1)  (19.2)  (60.6) | 23  17  32 | (31.9)  (23.6)  (44.4) | 20  24  97 | (14.2)  (17.0)  (68.8) | 0.285 |
| **Comorbidities*** | Thyroid dysfunction | 65 | (30.5) | 36 | (50.0) | 29 | (20.5) | <0.01 |
|  | Cardiac dysfunction | 9 | (4.2) | 5 | (6.9) | 4 | (2.8) | 0.17 |
|  | Gonadal dysfunction | 112 | (52.6) | 46 | (63.9) | 76 | (53.9) | 0.189 |
|  | Pulmonary dysfunction | 44 | (20.7) | 14 | (19.4) | 30 | (21.3) | 0.853 |
|  | Precocious puberty | 14 | (6.6) | 9 | (12.5) | 5 | (3.5) | 0.018 |

*Variables were assessed at the time of short stature diagnosis or at initiation of GH therapy.

Percentages are calculated using the total number of patients in each group as the denominator. Missing data are indicated where applicable.

GH; Growth hormone, IQR; Interquartile range, HCT; Hematopoietic cell transplantation, RT; Radiation therapy, TBI; Total body irradiation, CSI; Craniospinal irradiation, CRT; Cranial radiotherapy, BU; Busulfan, MEC; Melphalan/Etoposide/Carboplatin, TEPA; Thiotepa, MAC; myeloablative conditioning

**Supplementary TABLE 7. Univariable analysis of complications associated with GH therapy**

| **Variables** | **Category** | **Total*** | **Slipped capital femoral epiphysis** | | | **Secondary malignancy** | | | **Relapse** | | |
| --- | --- | --- | --- | --- | --- | --- | --- | --- | --- | --- | --- |
|  |  | **No.** | **No.** | **%** | ***p* value** | **No.** | **%** | ***p* value** | **No.** | **%** | ***p* value** |
| GH therapy | Yes | 72 | 4 | (5.5) | 0.183 | 15 | (20.1) | 0.16 | 8 | (11.1) | 0.312 |
|  | No | 141 | 2 | (1.4) |  | 18 | (12.7) |  | 10 | (7.1) |  |
| TBI | Yes | 115 | 5 | (4.3) | 0.221 | 26 | (22.6) | <0.01 | 7 | (6.1) | 0.22 |
|  | No | 98 | 1 | (1.0) |  | 7 | (7.1) |  | 11 | (11.2) |  |
| BU | Yes | 44 | 1 | (2.3) | 1 | 6 | (13.6) | 0.818 | 6 | (13.6) | 0.219 |
|  | No | 169 | 5 | (3.0) |  | 27 | (16) |  | 12 | (7.1) |  |
| Type of HCT | Allogenic | 144 | 4 | (2.8) | 1 | 20 | (13.9) | 0.419 | 12 | (8.3) | 1 |
|  | Autologous | 69 | 2 | (2.9) |  | 13 | (18.8) |  | 6 | (8.7) |  |
| Age at HCT | ≥10 years | 23 | 0 | (0.0) | 1 | 1 | (4.3) | 0.217 | 2 | (8.7) | 1 |
|  | <10 years | 190 | 6 | (3.2) |  | 32 | (16.8) |  | 16 | (8.4) |  |
| Chronic GVHD | Yes | 56 | 2 | (3.6) | 0.654 | 8 | (14.3) | 0.833 | 5 | (8.9) | 1 |
|  | No | 157 | 4 | (2.5) |  | 25 | (15.9) |  | 13 | (8.3) |  |
| Steroid | Yes | 40 | 1 | (2.5) | 1 | 4 | (10.0) | 0.463 | 3 | (7.5) | 1 |
|  | No | 169 | 5 | (3.0) |  | 28 | (16.6) |  | 15 | (8.9) |  |

* The analytic cohort consisted of 213 patients, including those without available final height data.

GH; Growth hormone, TBI; Total body irradiation, BU; Busulfan, HCT; Hematopoietic stem cell transplantation, GVHD; Graft-versus-host disease
