# Supplementary material for: Growth hormone therapy after hematopoietic cell transplantation in childhood: a nationwide survey and longitudinal cohort study
Source: Front Endocrinol (Lausanne). 2026 May 20;17:1831711. doi: 10.3389/fendo.2026.1831711 (PMC13229625; doi:10.3389/fendo.2026.1831711)
Supplement: Supplementary file 2 [file DataSheet2.docx]

# Supplementary Appendix. Participating institutions and institutional representatives

*Institutions are listed in alphabetical order.*

| **Institution** | **Institutional representative** |
| --- | --- |
| Abashiri Kosei Hospital | Hiroki Kajino |
| Asahikawa Medical University Hospital | Takehiro Sarashina |
| Chiba Children’s Hospital | Hidemasa Ochiai |
| Chiba University Hospital | Moeko Hino |
| Defense Medical College Hospital | Kosuke Imai |
| Dokkyo Medical University Hospital | Mayuko Okuya |
| Ehime Prefectural Central Hospital | Kozo Nagai |
| Ehime University Hospital | Kyoko Moriya |
| Fukui University Hospital | Toshihiro Fujiki |
| Gifu Municipal Hospital | Yoshifumi Yokoyama |
| Gifu University Hospital | Michio Ozeki |
| Gunma Children’s Medical Center | Hirohide Kawasaki |
| Hamamatsu University School of Medicine Hospital | Kimiyoshi Sakaguchi |
| Hirosaki University Hospital | Akie Kobayashi |
| Hokkaido University Hospital | Shuntaro Morikawa |
| Hyogo Prefectural Kobe Children’s Hospital | Daiichiro Hasegawa |
| Ibaraki Children’s Hospital | Keisuke Kato |
| Iwate Medical University Hospital | Shoko Miura |
| Japanese Red Cross Aichi Medical Center Nagoya First Hospital | Nao Yoshida |
| Kanazawa University Hospital | Toshihiro Fujiki |
| Kobe University Hospital | Nobuyuki Yamamoto |
| Kurashiki Central Hospital | Seishiro Nodomi |
| Kyoto City Hospital | Hiroyuki Ishida |
| Kyoto Prefectural University of Medicine Hospital | Shinya Osone |
| Maizuru Medical Center | Kohei Mitsuno |
| Mie University Hospital | Masahiro Hirayama |
| Miyagi Children’s Hospital | Atsushi Sato |
| Miyazaki University Hospital | Hiroshi Moritake |
| Nagoya University Hospital | Yoshiyuki Takahashi |
| Nara Medical University Hospital | Taku Ishihara |
| National Hospital Organization Nagoya Medical Center | Naoko Maeda |
| Nihon University Itabashi Hospital | Katsunori Shimozawa |
| Osaka International Cancer Institute | Yoshiko Hashii |
| Sapporo Medical University Hospital | Masaki Yamamoto |
| Shizuoka Cancer Center | Rieko Taniguchi |
| Shizuoka Children’s Hospital | Kenichiro Watanabe |
| Teikyo University Hospital | Mitsuteru Hiwatari |
| The University of Tokyo Hospital | Motohiro Kato |
| Tokushima University Hospital | Kazumi Okamura |
| Tokyo Metropolitan Children’s Medical Center | Masanaka Sugiyama |
| University of Tsukuba Hospital | Hiroko Fukushima |
| Yamagata University Hospital | Tetsuo Mitsui |
| Yokohama City University Hospital | Masanobu Takeuchi |
| Yokohama City University Medical Center | Kentaro Shiga |
